# Inflammation and Wnt Signaling: Target for Immunomodulatory Therapy?

**DOI:** 10.3389/fcell.2020.615131

**Published:** 2021-02-04

**Authors:** Imen Jridi, Kirsten Canté-Barrett, Karin Pike-Overzet, Frank J. T. Staal

**Affiliations:** Department of Immunology, Leiden University Medical Center, Leiden, Netherlands

**Keywords:** Wnt, TCF (T-cell factor), therapeutic targets, inflammatory disease, IL1b

## Abstract

Wnt proteins comprise a large family of highly conserved glycoproteins known for their role in development, cell fate specification, tissue regeneration, and tissue homeostasis. Aberrant Wnt signaling is linked to developmental defects, malignant transformation, and carcinogenesis as well as to inflammation. Mounting evidence from recent research suggests that a dysregulated activation of Wnt signaling is involved in the pathogenesis of chronic inflammatory diseases, such as neuroinflammation, cancer-mediated inflammation, and metabolic inflammatory diseases. Recent findings highlight the role of Wnt in the modulation of inflammatory cytokine production, such as NF-kB signaling and in innate defense mechanisms as well as in the bridging of innate and adaptive immunity. This sparked the development of novel therapeutic treatments against inflammatory diseases based on Wnt modulation. Here, we summarize the role and function of the Wnt pathway in inflammatory diseases and focus on Wnt signaling as underlying master regulator of inflammation that can be therapeutically targeted.

## Introduction

Inflammation is a complex biological defense against harmful pathogens or toxins. Inflammation is also a response to physical, ischemic, or other types of tissue damage. This protective inflammatory response causes cellular changes and immune responses that result in clearance of the initial cause of the injury and removal of necrotic cells, followed by cellular proliferation and growth at the site of the infected tissue. Together with other leukocytes, macrophages generate high levels of reactive oxygen and nitrogen species, and in collaboration with T lymphocytes, they release cytokines, such as TNFα, in order to fight infection. Various inflammatory mediators, including vasoactive amines and peptides, eicosanoids, proinflammatory cytokines (interferons, TNFs, IL1, IL6), and acute-phase proteins, are key contributors in creating such a microenvironment and are involved in cellular transformation, survival, and proliferation (Singh et al., 2019). Over the last few years, it has become apparent that Wnt signaling is also involved in the modulation of immune responses during inflammation. As examples, the complex β-catenin/TCF-1 is able to initiate Th2 differentiation by activating GATA3 transcription (Notani et al., 2010). On the contrary, CD4^+^CD25^+^ Tregs expressing stable β-catenin inhibit inflammation more effectively due to their superior ability to survive (Ding et al., 2008). These data suggest that Wnt signaling may play an important role in regulating inflammation and might be an interesting therapeutic target to control inflammation. In this review, we summarize the different Wnt pathways and the molecular mechanisms underlying each one of them. We illustrate its specificity and its functions in physiological and pathological conditions. Because of the rapid progress in the field, Wnt function is often reviewed in the context of hematopoiesis (Staal et al., 2008). In this review, we focus on Wnt function in immune response modulation during inflammatory disease.

## Overview of Wnt Signaling

### Wnt Pathway

**Figure 1:**
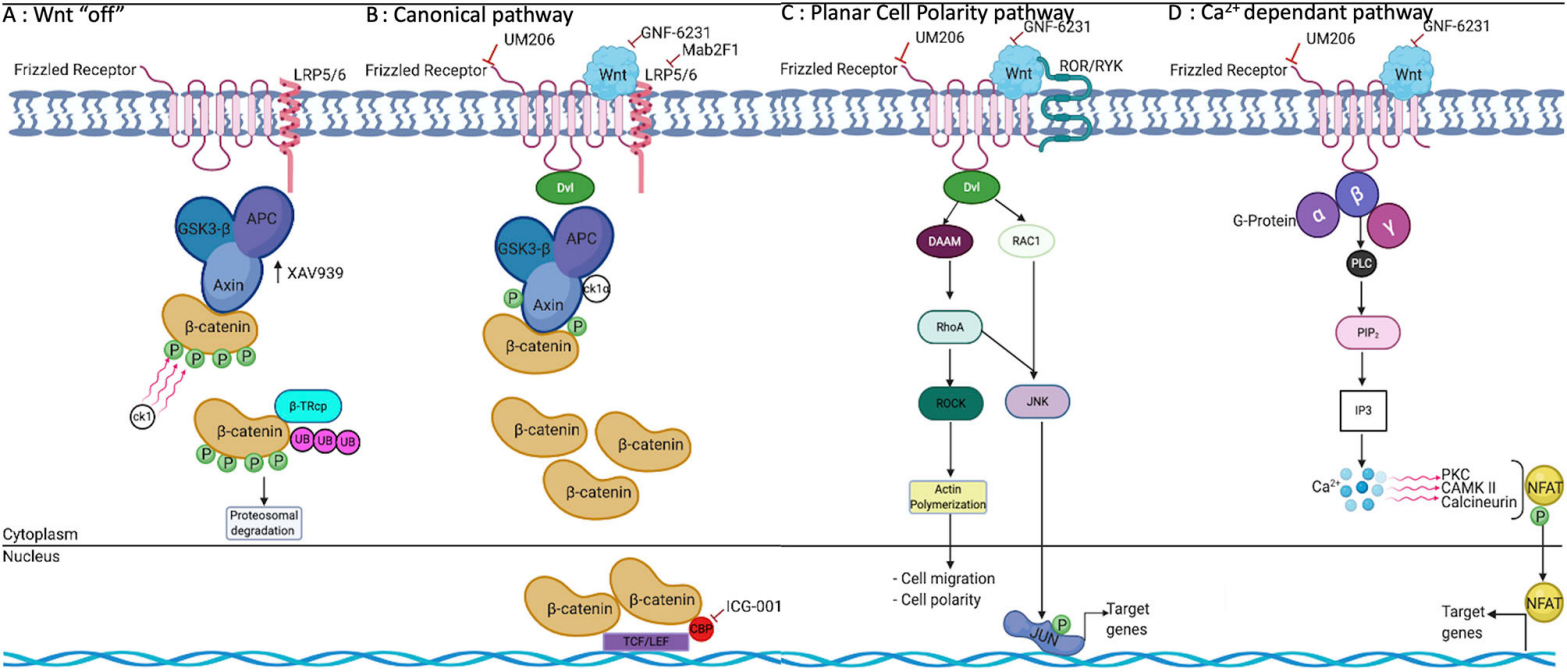
Wnt signaling pathways and their modulation by pharmacological molecules. **(A)** In the absence of Wnt, β-catenin is phosphorylated and degraded by the destruction complex. **(B)** β-catenin is accumulated in the cytoplasm and translocated to the nucleus, where it binds to TCF/LEF proteins, leading to the transcription of target genes. **(C)** PCP is one of two major non-canonical pathways and is involved in cell migration, motility, and polarity. **(D)** The Ca^2+^-dependant pathway involves the release of intracellular free calcium, which regulates a number of calcium-dependent signaling molecules, including PKC, CamKII, and the phosphatase calcineurin, which dephosphorylates NFAT. TCF, T cell factor; LEF, lymphoid enhancer factor; PKC, protein kinase C; CamKII, calmodulin kinase II; NFAT, Nuclear Factor of Activated T cells; PLC, Phospholipase C; IP3, inositol tri phosphate.

The evolutionary conserved Wnt pathway is a complex signaling network involving multiple molecular mediators and targets. To date, 19 distinct Wnt proteins, 10 frizzled receptors (FZD), and several co-receptors have been identified in mammals (Jia et al., 2008; Pereira et al., 2009; Jridi et al., 2020). The Wnt signaling machinery entails two highly specialized pathways: the canonical pathway, commonly referred to as the β-catenin-dependent pathway, and the non-canonical pathway referred to as the β-catenin independent pathway (Figure 1). Briefly, in the canonical pathway, Wnt proteins bind to their complex receptor FZD/LRP5/6 (low-density lipoprotein receptor related protein), preventing proteasomal degradation of β-catenin. Subsequently, β-catenin is translocated to the nucleus, where it binds to one of four transcription factors: T cell factor (TCF 1, 3, 4) or the lymphoid enhancer factor (LEF). Once these DNA-binding proteins are activated, several target genes are expressed, including cyclin-D1, c-Myc, and Axin-2 and, in muscle cells, Myo-D (Guo et al., 2008; Pereira et al., 2009; Staal and Arens, 2016; Meyer and Leuschner, 2018; Garcia de Herreros and Dunach, 2019; Jridi et al., 2020; Li et al., 2020). Conversely, in the absence of the Wnt ligand, the destruction complex, composed by Axin − 1 and/or−2, APC, GSK3β, CK-1, and β-TrCP remains active and phosphorylates β-catenin on its conserved ser and thr residues in the N-terminal domain (Figure 1). Consequently, phosphorylated β-catenin is targeted for ubiquitination and proteasomal degradation. As a result, the pool of β-catenin in the cytosol is depleted, and its nuclear translocation is blocked, which ultimately restrains Wnt downstream gene transcription (Shi et al., 2017; Ackers and Malgor, 2018; Meyer and Leuschner, 2018; Garcia de Herreros and Dunach, 2019; Aamir et al., 2020). A hallmark of the non-canonical signaling pathway is its β-catenin independent action, and it usually involves changes in cell motility or shape. The diversity and physical availability of receptors and co-receptors makes non-canonical Wnt signaling highly cell type–specific (Shi et al., 2017; Ackers and Malgor, 2018; Garcia de Herreros and Dunach, 2019; Aamir et al., 2020). In any case, non-canonical Wnt signaling is initiated by the binding of non-canonical Wnt to FZD as well as to a variety of co-receptors. Based on the diversity of co-receptors, five pathways were identified. Planar cell polarity (PCP) results in FZD/Dvl-mediated activation of the small GTPases RAC and RHOA. Wnt/Ca^2+^ signaling is also initiated by FZD/Dvl binding, but it mediates PLC activation and an increased level of cytosolic Ca^2+^, which activates calmodulin/calmodulin-dependent kinase II and NFAT-regulated transcription (Figure 1). The other non-canonical pathways involve recognition of distinct Wnt ligands by cognate FZD/ROR receptors, FZD/Ryk, and FZD/JNK (Staal and Arens, 2016; Ackers and Malgor, 2018; Houschyar et al., 2018; Meyer and Leuschner, 2018; Ljungberg et al., 2019; Aamir et al., 2020).

### Specificity of Wnt Signaling

Even though Wnt signaling has been explored extensively, the specificity of Wnt interactions with their ligands remains poorly understood. This could be explained by the difficulty of studying the large variety of receptors, co-receptors, and adapter molecules involved in canonical and non-canonical pathways. Additionally, the course of Wnt-FZD signaling is dictated to a great extent by the relative densities of different Wnt ligands in the intracellular milieu (Steinberg, 1944; Wong and Adler, 1993; Boutros et al., 2000). It is rather interesting to note that LRP5/6 is the diverging point of different Wnt-FDZ signaling pathways. LRP5/6 is composed of 3 segments: P1E1-P2E2, P3E3-P4E4, and LDLR type A. P1E1 has an affinity to Wnt1, Wnt2, Wnt2b, Wnt6, Wnt8a, Wnt9a, Wnt9b, and Wnt10a, whereas P3E3 interacts with Wnt3 and Wnt3a (He et al., 2004; Bourhis et al., 2010). Besides this, a complex Wnt3a/Wnt9b/LRP6 has been detected, suggesting that a single LRP6 or LRP5 may simultaneously interact with different Wnt proteins. This variable affinity profile could lead to the synthesis of different functional protein complexes in the divergent Wnt pathway (Bourhis et al., 2010). Dvl, another branching point of Wnt signaling, could adopt different conformations based on the level of phosphorylation, leading to the expression and the activation of different proteins (Garcia de Herreros and Dunach, 2019). Thus, the final outcome of Wnt signaling may depend on the combined impacts of several ligand–receptor junctions and different pathways (Sen, 2005; MacDonald and He, 2012; Garcia de Herreros and Dunach, 2019; Jridi et al., 2020).

### Transport of Wnt Proteins Between Cells

An important but less studied aspect of Wnt signaling is the mechanism of the intercellular transport of Wnt proteins after secretion. However, a few specific Wnt exporter models have been proposed. Some studies suggest that Wnt could be transported by a thin filopodial process called cytonemes (Ramírez-Weber and Kornberg, 1999), and others propose the idea that Wnt could be transported outside the cell via a vesicle-based transport called argosome (Greco et al., 2001). All these modes of vesicle-mediated transport of Wnt between cells have been characterized in *Drosophila*, but such a mechanism has not been identified yet in mammals. Nevertheless, it seems likely that heparin sulfate proteoglycans (HSPGs) that act as co-receptors for Wnts on the extracellular matrix of many connective tissues also coordinate Wnt transport between cells (Logan and Nusse, 2004; Yamazaki et al., 2016). Interestingly, many of these extracellular matrix components themselves are targets of canonical Wnt signaling (Staal et al., 2004; Piccolo et al., 2014; Noguchi et al., 2018).

### Functional Importance of Wnt Signaling

It is well-established that Wnt signaling is a key regulator of a variety of biological processes ranging from embryonic development and tissue homeostasis to stem cell function. Wnt proteins function as proliferation-inducing growth factors and may also affect cell fate decisions, apoptosis, and quiescence (Clevers, 2006; Aoyama et al., 2007; Staal and Arens, 2016; Xiao et al., 2016). Besides this, Wnt proteins provide cytokines/chemokines, adhesion, and extracellular matrix molecules that promote the growth, differentiation, and survival of several cell lineages and types (Staal et al., 2004; Piccolo et al., 2014; Ackers and Malgor, 2018; Noguchi et al., 2018). It is, thus, not surprising that dysregulation of Wnt signaling is implicated in a multitude of diseases including fibrosis, bone density disorders, obesity, type 2 diabetes, non-alcoholic fatty liver disease, chronic kidney disease, cardiovascular and neurodegenerative diseases, and variety of inflammatory diseases. Importantly, in many tissues and best established in the hematopoiesis and lymphocyte development, canonical and non-canonical Wnt signaling have opposite effects. It, therefore, is important to discriminate between these different outcomes when discussing Wnt as regulator of inflammatory processes (Malhotra et al., 2008; Luis et al., 2010; Famili et al., 2015). In the next section, we describe the cross-talk between Wnt and inflammatory pathways.

## Connection Between Wnt Signaling and Inflammatory Pathways

As mentioned, Wnt signaling is involved in the development and progression of several diseases. This could only be explained by the increase of cross-talk between Wnt signaling and several other pathways, such as TCR and BCR signaling. TCR signaling is known for its ability, in cooperation with CD28, to inhibit GSK3β kinase activity, leading to an increase in cytoplasmic levels of β-catenin. Therefore, it is not surprising that the stimulation of human primary cells with agonist antibodies to TCR leads to the upregulation of β-catenin (Lovatt and Bijlmakers, 2010). Interestingly, the increase of β-catenin was strongly induced in protein level but not in mRNA level, suggesting that TCR signaling can affect β-catenin after transcriptional events or maybe during protein translation and its stability. It is worth noting that CD28 cooperates with PI3K to activate AKT, leading to GSK3β phosphorylation, its deactivation, and, thus, β-catenin accumulation (Lovatt and Bijlmakers, 2010). Consistently, exposition of human primary T cells to LY294002, a PKI3 inhibitor, leads to the decrease of GSK3 phosphorylation and β-catenin level. However, all these effects were recovered upon the stimulation of cells with the potent PKC activator PDBu. Taken together, these data suggest that TCR signaling, via PI3K as well as PKC, has the ability to phosphorylate GSK3β and, thus, stabilize β-catenin (Lovatt and Bijlmakers, 2010). Unexpectedly, this stabilized β-catenin was not always in the N-terminally dephosphorylated form and, therefore, did not lead to increase in Wnt/TCF-dependent transcription. Hence, different pools of β-catenin may exist that do not all lead to synergistic activation of Wnt-mediated transcriptional events (Lovatt and Bijlmakers, 2010). This is consistent with other reports showing that PKC by phorbol esters in T cells leads to increased nuclear β-catenin and not to increased Wnt signaling (Atcha et al., 2007). These studies serve as examples that increased components of the Wnt pathway do not always indicate their interaction with other pathways.

It has been reported that Wnt signaling is activated in different human cancers. Indeed, diffuse large B cell lymphoma (DLBCL) cells were able to secrete Wnt3a and activate Wnt signaling. This could be explained by the fact that the tumor microenvironment contributes to β-catenin accumulation in the cytoplasm. However, besides tumor or inflammatory microenvironments (Koch et al., 2014), other parallel mechanisms are involved in β-catenin stabilization. As in many oncogenic and inflammatory pathways, β-catenin was detected in DLBCL-CARMA1 mutant (L225LI), indicating that active mutant CARMA1 leads to the expression and the activation of β-catenin.

A large number of studies indicate an interaction between NF-κB signaling and the canonical Wnt pathway; most often this is a negative regulatory interaction in which Wnt signaling inhibits NF-κB activity (Rodriguez-Pinilla et al., 2005; Sun et al., 2005; Duan et al., 2007; Du et al., 2009; Moreau et al., 2011; Liu et al., 2013; Ma et al., 2015). NF-kB signaling controls a large number of diverse target genes, such as cytokines, chemokines, growth factors, immune receptors, transcription factors, and repressors of apoptosis (Winston et al., 1999). Hence, NF-κB function is a crucial regulator of local and systemic inflammation. In simple terms, the NF-kB pathway consists of a cytoplasmic bound transcription factor complex made up of two proteins (either homodimer or heterodimer) of the Rel family of proteins that are retained in the cytoplasm by an inhibitor protein termed I-kB. Upon phosphorylation by I-KB kinases, the phosphorylation leads to a breakdown of the inhibitor, releasing NF-kB to migrate to the nucleus and activate its target genes. In some reports, β-catenin was found to complex with NF-KB, leading to a decrease in NF-κB DNA binding and NF-KB-dependent transcription (Winston et al., 1999). Other mechanisms also have been proposed, including GSK-3 inhibition, leading to a reduction in NF-κB activity. It should be noted that stimulatory effects of β-catenin on NF-κB activity have also been reported. This can be explained by the similar protein motifs in IkB and b-catenin for binding Ubiquitin ligases. β-TrCP is an E3 ubiquitin ligase resulting in the ubiquitination and subsequent degradation of both β-catenin and IκB-α (Winston et al., 1999). Wnt signaling stabilizes β-catenin and can elevate β-TrCP expression, resulting in enhanced degradation of IκB-α and, therefore, enhanced NF-κB transactivation.

## The Role of Wnt in Innate and Adaptive Immunity During Inflammation

### Lung Inflammation

The impact of Wnt/β catenin and non-canonical pathways in inflammatory and immunological responses in the lung, such as allergic airway inflammation, have been analyzed in a number of studies returning varying results. The selective deletion of Wnt10b in mice sensitized with house dust mites (HDMs) rendered the mice more sensitive to inflammation. This was associated with increased numbers of inflammatory cells, including activated macrophages, with a predominance of eosinophils in the lung and also a significant increase of the Th2 response as indicated by an increase of the specific markers IL-4, IL-3, and GATA3 as well as the chemoattractant CCL2 involved in Th2 polarization. It is interesting to note that genetic deletion of Wnt10b in HDM-sensitized mice enhanced the response of T cell effectors, shown by the increase of CD44^hi^ CD62L^lo^ CD4^+^, CD8^+^, and CD69^hi^ CD11a^hi^ but had no effect on CD25^+^FoxP3^+^Treg cells, suggesting that the Wnt pathway might direct the Th2 but not the Treg response during inflammation (Trischler et al., 2016). Consistently, in silicosis induced by inhalation of silica mineral dust, the blockade of β-catenin with a specific shiRNA exhibited significantly aggravated inflammation. This was associated with the accumulation of inflammatory cells, particularly neutrophils and lymphocytes, and the production of pro-inflammatory cytokines such as IL-1β, TNFα, and IL-6 in the bronchoalveolar lavage fluid (BALF). Th17 response has been implicated during silica-induced inflammation in mice by increasing the expression of IL-6, IL-17A, IL-21, and RORγT and the percentage of CD4^+^IL-17A^+^ T cell population in lymph nodes. Similarly, in the same experimental approach, the blockade of the Wnt/β-catenin pathway enhanced Th1 response by increasing the expression of IFNγ and T-bet and the proliferation of the CD4^+^ IFNγ^+^ T cell population. These findings suggest that the function bias of silica-induced inflammation, in the absence of β-catenin, is toward fostering Th1 and Th17 response at the detriment of Treg cells by decreasing FoxP3, TGFβ, and IL-10 and of Th2 response by reducing GATA3, IL-4, and IL-3 levels (Dai et al., 2016). Nevertheless, in an OVA-expressing inflammation model, mice treated with LiCl (agonist of the canonical Wnt signaling that can inhibit GSK3β activity and thereby stabilize free cytosolic β-catenin effectively) showed a significant decrease of Th2 cytokines, such as IL-4 and IL-5, abolishing the Th2 response (Reuter et al., 2014). Also, there are strong indications that Wnt signaling can affect Treg function directly (van Loosdregt and Coffer, 2018) by reducing Treg-cell–mediated suppression *in vitro* and *in vivo*. These divergent results highlight the variety of immunological responses of the Wnt/β-catenin pathway during inflammation. Such variability could be likely driven by endogenous modulation of Wnt ligands and their signaling events by specifically targeting the downstream genes at the gDNA level (genetic perturbation) or by the use of small molecule inhibitors or activators or by exogenous modulation through DNA engineering (recombinant Wnt) conditioned media by adding Wnt regulators to cell culture (Ljungberg et al., 2019). However, it is really important to outline whether factors specific to the pathophysiological context (e.g., immune regulatory molecules, cytokines milieu) could be involved in the apparently opposing responses of Wnt during inflammation (Th2 response vs. Treg response and vice versa).

### Neuroinflammation

The dysregulation of the Wnt/β-catenin pathway occurs in multiple neurodegenerative diseases, including Alzheimer (De Ferrari et al., 2014), Parkinson (Le Grand et al., 2015), and multiple sclerosis (Giacoppo et al., 2017). Their expression in immune-like cells of the CNS, including macrophages/microglia and astrocytes, as well as in oligodendrocytes suggests that they contribute to inflammation-driven brain repair or damage. DC-specific deletion of LRP5/6 or β-catenin leads to the increase of experimental autoimmune encephalomyelitis (EAE) and intense demyelination. Mechanistically, the genetic deletion of LRP5/6 or β-catenin in DC ensures a marked increase in leukocyte infiltration accompanied by the increase of pro-inflammatory cytokines, such as IFNγ, IL-17A, IL-22, IL-6, TNFα, IL-1β, IL-12p40, and IL-12p70, in the draining lymph node and CNS and the decrease of anti-inflammatory cytokine production (IL-10), indicating the critical role of the Wnt/β-catenin pathway in DC for limiting autoimmune CNS pathology. Furthermore, the absence of the Wnt pathway component during EAE reduced IL10^+^CD4^+^ regulatory cells Treg response and increased the Th1 and Th17 inflammatory response (increase of IFNγ^+^IL17^+^, TNFα^+^ CD4^+^, IFNγ^+^, and TNFα^+^ CD8^+^ T cells). This suggests a regulatory role of the canonical Wnt pathway during ongoing neuroinflammation in which activation of this pathway regulates the differentiation of naïve CD4^+^ T cells into Th1 and Th17 through regulation of DC-specific cytokine production and limitation of uncontrolled differentiation. Moreover, prophylactic and therapeutic treatment of EAE mice with a β-catenin agonist restores the injury by significantly delaying EAE onset and by markedly reducing its severity. This was explained by the reduction of the expression of pro-inflammatory cytokines and the increase of anti-inflammatory cytokines. It seems that β-catenin activation during EAE limits CNS pathology via the suppression of Th1 and Th17 response, the increase of Treg cells frequency, and the promotion of the regulatory response, suggesting that immunotherapy targeting Wnt/β-catenin could represent a promising therapeutic approach for clinical management of multiple sclerosis (Suryawanshi et al., 2015). However, other studies reveal that activation of the Wnt pathway could aggravate inflammation. The effect of β-catenin expression appears dose-dependent: transgenic mice with biallelic β-catenin overexpression develop lethal leukemia by blocking T cell development in the thymus at the immature CD4^+^CD8^+^ double positive stage. In contrast, transgenic mice with monoallelic β-catenin overexpression revealed a moderate increase in the frequency of infiltrating CD4^+^ and CD8^+^ T cells and their “partial activation” accompanied by accumulation of T cells at the double negative (DN) stage, increase of their proliferation, and the blockade of their apoptosis. In addition, a very strong decrease of naïve T cell marker CD62L concomitant with an enhancement of activation memory marker CD44 and CD69 on CD4^+^ and CD8^+^ T cells in the thymus and in the splenic and peripheral lymph node was detected. β-catenin overexpression recruits activated T cells to initiate a Th1-type inflammatory cascade by increasing IFNγ, IL-2, and TNFα expression and activates involving microglia and astroglia, leading to neurologic abnormalities and motor impairment in mice (Sorcini et al., 2017). Taken together, dysregulation of the Wnt/β-catenin pathway could be implicated in human CNS inflammatory and other autoimmune/inflammatory diseases, but potential detrimental effects of overactivation of the Wnt pathway should also be considered. Therefore, further study of comprehensive characterization and molecular machineries about the involvement of this pathway in human inflammatory and neuro-degenerative diseases is needed.

### Colon and Intestinal Inflammation

The Wnt signaling pathway plays a crucial role in intestine development and in maintaining gut homeostasis (Greco et al., 2001). Aberrant Wnt signaling was recently detected in multiple intestinal inflammatory diseases, including inflammatory bowel disease (IBD) and IBD-associated colon cancer (Clevers, 2006). Nevertheless, β-catenin activity in myeloid cells has emerged as a rheostat in immune regulation and tolerance, specifically in *in vivo* models for auto-immunity, gut mucosal homeostasis, and cancer.

Chemical toxins, such as locally applied dextran sodium sulfate (DSS) provides an experimental model of tissue injury and intestinal inflammation. Recent studies highlight that LRP5/6 are still expressed in colonic DC and macrophages in this model. Accordingly, selective deletion of LRP5/6 in CD11c^+^ APC rendered mice highly susceptible to DSS. This was related to the elevated expression of pro-inflammatory cytokines, such as IL-1β, IL-6, IFN-γ, and IL-17; the reduction of anti-inflammatory cytokine expression, such as IL-10 and IL-22; the differentiation of naïve CD4^+^ T cells into Th1 and Th17; and the decrease of FOXP3 Treg differentiation. These data suggest that the functional bias, in the absence of LRP5/6, has been to enhance the effector response Th1 and Th17 and to suppress Treg response. However, mice with LRP5/6-deficient CD11c^+^ cells that express a stabilized form of β-catenin had a reduction of the severity of DSS-induced colitis as marked by reduced inflammation-associated tissue injury and reduced weight loss. Inflammatory cytokines were reduced, and mRNA levels of IL-10 was increased (Swafford et al., 2018). Similar experimental approaches confirmed the anti-inflammatory role of β-catenin in other mouse models. DCs lacking β-catenin in mice treated with DSS showed a severe inflammatory response associated with an increase in inflammatory cell infiltration, edema, epithelial cell hyperplasia, and loss of goblet cells, indicating the critical role of β-catenin signaling in DC in the regulation of intestinal homeostasis and the tolerogenic state. It is interesting to note that β-catenin-deficient DCs resulted in a low frequency of Treg cells and a high frequency of Th17 and Th1, indicating that β-catenin signaling in DC is critical for maintaining the balance between Treg cells and CD4^+^ T effector populations. The result from β-catenin-deficient DCs was confirmed by DC treatment with LiCl, which induces higher frequencies of Treg cells (Manicassamy et al., 2010).

The aforementioned studies support the view that activation of β-catenin in intestinal DCs programs them to induce Treg cells. In addition to the results from the mice overexpressing β-catenin in LRP5/6-deficient CD11c^+^ DCs as discussed above, high levels of β-catenin in T cells can cause chronic T cell activation by the expression of several activation markers including CD69, CD122, and NK62D and causes Th17 commitment by the expression RORγT, the signature transcription factor of the Th17 lineage. The activation of the latter transcription factor is responsible for Treg dysfunction, which loses the anti-inflammatory functions and contributes to systemic inflammation, colitis, and polyposis (Keerthivasan et al., 2014).

### Hepatic Inflammation

The liver is an immunologic organ in which the main non-immunologic roles are metabolism, body detoxification, and nutrient storage. The liver shares both tolerogenic and immunogenic properties with the immune system in its composition and features, such as the ability to induce immune tolerance, production of innate immunity proteins, and hematopoiesis in the fetal liver (Gao, 2016; Tan et al., 2020). The liver is continuously exposed to a wide variety of antigens, bacteria, and endotoxins from the gastrointestinal tract (Gao, 2016; Tan et al., 2020). Thus, under microbial attack, it easily switches from the tolerogenic ground state to immunogenic response. This swift transition is due to the hepatic dendritic cells (HDC). In order to maintain tolerance and integrity under a steady-state condition, HDCs preserve their immature phenotype by expressing low MHCII and costimulatory molecules and producing anti-inflammatory mediators. In an autoimmune hepatitis (AIH) mouse model, HDC were converted into an immunogenic state marked by the maturation and the activation of CD40, CD80, CD86, CCR7, and MHCII; by the increase of inflammatory cytokines IL-1a, IL-2, IL-12a, IL-12b, and IFNγ; and by the decrease of the regulatory mediator IL-10 and TGF-β. Surprisingly, the level of β-catenin and its target genes Axin2 and TCF-4 in decreased in AIH-HDC. Chemical activation of β-catenin in HDC from concanavalin A (ConA)-induced AIH mouse model resulted in the reversion to an immunoregulatory phenotype with a low level of CD80, CD86, CCR7, IL-2, IL-12a, and IL-12b and a high level of PD-L1, IL-10, and TGF-β, indicating that reactivating Wnt/β-catenin signaling pathway can restore the immunoregulatory phenotype of HDCs. Consistent with that, the vaccination of ConA-induced AIH mice with Wnt ligand–pretreated DCs showed the decrease of the liver injury associated with the decrease of pro-inflammatory cytokines (IL-2, IL-12, and IFNγ), the increase of anti-inflammatory cytokines (IL-10 and TGF-β), and the reduction of the number of intrahepatic CD4^+^ and CD8^+^ T cells. These effects suggest that Wnt ligands enable HDC to eliminate the activation of T cells and govern Treg differentiation, thereby ameliorating the severity of AIH. These studies show the potently suppressive effect on AIH inflammation, suggesting that Wnt modulation could be a therapeutic strategy for AIH patients (Tan et al., 2020).

### Systemic Inflammation in Tumor and Other Disease Models

Fibrosis is a common pathological mechanism to promote wound healing of chronic inflammation in different organs of the body, including liver, kidney, heart, and lung. It leads to organ decomposition and destruction (Wynn and Ramalingam, 2012). Pulmonary fibrosis results from the development and the progression of chronic inflammation associated with infection, air pollution, cigarette smoking, and cancer therapy (Selman et al., 2016). During airway damage, the upregulation and activation of β-catenin in alveolar epithelial type II cells induces the activation and remodeling of interstitial fibroblasts, which could and, in this case, does lead to pulmonary fibrosis (Wynn and Ramalingam, 2012). Besides this, the activation of canonical Wnt/β-catenin in pulmonary endothelial cells induces the development of perivascular fibroblasts into myofibroblast-like cells. The latter accumulate extracellular matrix and increase tissue stiffness, thus causing pulmonary fibrosis (Andersson-Sjoland et al., 2016; Cao et al., 2016). The crucial role of Wnt/β-catenin during liver pathophysiology is associated with chronic inflammation, such as hepatocyte proliferation, liver fibrosis, and tumorigenesis. HBV is a virus that infects hepatocytes and induces them to produce several viral proteins, such as HBx. HBx induces the upregulation and activation of EBCAM, MYC, NF-kB, and β-catenin in hepatic cells. The activation of β-catenin, through RSPO, plays a critical role in the process of hepatic stellate cell activation and liver fibrosis promotion (Yin et al., 2016). Indeed, β-catenin inhibitor PRI-724 was able to prevent HCV-related liver fibrosis by abolishing β-catenin activation (Tokunaga et al., 2017).

Accumulation of visceral adiposity is another factor associated with systemic inflammation. It is shown that Wnt5a promotes inflammation and insulin resistance in adipose tissue using animal models (Ouchi et al., 2010; Fuster et al., 2015). Indeed, overexpression of Wnt/PCP signaling was observed in visceral adipose tissue (VAT). Interestingly, a marked upregulation of Wnt5a, its co-receptor ROR2, PTK7 (a membrane protein involved in Wnt5a/ROR2 binding and signaling in mammalian cells), and Wnt5a downstream genes was observed in VAT. The high level of Wnt5a was correlated with the increase of IL-6 secretion in VAT. Consistently, Wnt5a siRNA treatment induced the decrease of Wnt5a as well as IL-6, confirming the correlation between these proteins. IL-6 has been previously linked to metabolic alteration associated with visceral adiposity (Zuriaga et al., 2017) as well as CCL5 and IL7 produced by VAT (van der Weerd et al., 2012). Thus, these data highlight that overactivation of Wnt5a/PCP signaling in VAT contributes to the secretion of a high level of IL-6 and low-grade systemic inflammation under obesity conditions (Zuriaga et al., 2017).

In certain cancers, metastases can be so widespread that it leads to systemic disease. Such inflammatory processes contribute to further metastasis of tumor cells and, therefore, provide important therapeutic targets. Recent work from the de Visser lab indicates that, in a breast cancer model in which the p53 tumor suppressor gene has been deleted, cancer cells abundantly produce Wnt ligands that stimulate macrophages in the tumor microenvironment to produce IL-1β (Wellenstein et al., 2019). This drives inflammation by neutrophil recruitment and exacerbates disease in these mouse models. Strikingly, blocking Wnt production in tumor cells reduces the IL-1β production by macrophages, reducing systemic inflammation, and metastasis of tumor cells. Hence, in this model, Wnt functions as a master regulator of systemic inflammation, which can be therapeutically targeted.

## Wnt Signaling Modulation: A Novel Therapeutic Strategy to Dampen Inflammation?

In the models discussed, the activation of canonical Wnt signaling generally has a dampening effect on the immune response in the case of tissue-restricted inflammation, and in systemic inflammation, Wnt activation enhances inflammatory processes. Despite various implications of activated Wnt signaling in the pathophysiology of human inflammation, Wnt modulation using animal models has shown promising results in the amelioration of the inflammation-driven diseases. XAV939 (a Wnt/β-catenin pathway modulator) was able to significantly suppress the LPS effect on BEAS-2B human bronchial epithelial cells by decreasing pro-inflammatory gene expression, including IL-6, IL-8, TNFα, IL-1β, MCP-1, MMP-9, iNOS, and COX2. Although the expression of proinflammatory genes are increased, Ik-β is phosphorylated and degraded, followed by NF-kB release and its translocation from the cytoplasm to the nucleus. Even though XAV939 was able to significantly block Ik-β phosphorylation and NF-kB translocation from the cytoplasm to nucleus, this effect was rescued by β-catenin expression (Jang et al., 2019). Similarly, Wnt agonist treatment prior to hepatic ischemia/reperfusion (I/R) significantly reduces hepatocellular damage as the liver tissue was remarkably preserved and moderate hepatocellular edema was the only difference comparing the liver of sham-operated animals. This could be explained by the increase of the proliferative status of hepatocytes and the decrease of the number of apoptotic cells, suggesting that the principal activity of Wnt agonists could be the protection of hepatocytes from apoptosis during hepatic I\R recovery. Simultaneously, Wnt agonist administration led to a downregulation of pro-inflammatory markers (iNOS, Nitrotyrosine, IL-6) and the associated neutrophil recruitment. All this anti-inflammatory potential of Wnt agonists leads to a significant increase in the survival rate of the mice (Kuncewitch et al., 2013). Consistently, inhibition of Wnt signaling by Mab2F1 (a mouse monoclonal antibody against the E1E2 domain of LRP6) displayed therapeutic potential on diabetic retinopathy. Intravitreal delivery of Mab2F1 was shown to reduce vascular leakage, inhibit retinal inflammation, and ameliorate retinal neovascularization in the ocular inflammatory retinopathy model. This therapeutic effect was due to the inhibition of high-glucose-induced Wnt signaling and the suppression of inflammatory factors such as VEGF, ICAM-1, and TNF-α (Lee et al., 2014).

Wnt signaling is involved in heart disease on several levels: on the metabolic level by increasing cell sensitivity to insulin; on the cellular level by promoting smooth muscle cell proliferation; and on the structural and functional levels by causing fibrosis, sclerosis, atheroma formation, and hypertrophy (Saraswati et al., 2010). However, a select few molecules have shown promise to treat myocardial inflammation by targeting Wnt secretion or turnover of the β-catenin destruction complex (Willems et al., 2011). For example, GNF-6231, an inhibitor of both canonical and non-canonical Wnt ligands by the inhibition of Wnt palmitoylation, was able to limit pro-fibrotic myocardial injury and enhance recovery in preclinical models of myocardial infarction (Bastakoty et al., 2016). UM206 is a peptide characterized by its very high homology to Wnt3a and Wnt5a and its affinity to frizzled receptors, which inhibits Wnt signaling pathway transduction by blocking receptor occupancy. UM206 decreased heart failure and reduced the size of the myocardial infarction (MI) as well as increased the number of capillaries and decreasing myofibroblasts in the infarct area of the post-MI heart (Laeremans et al., 2011). Another small molecule that inhibits the interaction between β-catenin and CBP in the Wnt canonical signaling pathway is referred to as ICG-001 and was identified as a potent small molecule to treat heart disease. ICG-001 was able to regenerate myocardial cells by boosting the differentiation of epicardial progenitors, leading to the improvement of cardiac function in a rat model of myocardial infarction (Sasaki et al., 2013).

Recently, Wnt signaling became a therapeutic target for neurodegenerative diseases and neuro-inflammation, such as Parkinson disease. In an MPTP-based Parkinson model, Fasudil, a Rho kinase inhibitor, induced the protection of dopaminergic neurons from MTPT toxicity. This protective effect could be explained by its inhibitory effect exerted on phosphorylated GSK3β and, thus, the increase of β-catenin level accompanied by the increase of Wnt1/Fzd1 and the P110-PI3K/AKT pathway (Zhao et al., 2015). In addition, it has been reported that Wnt signaling activation ameliorates neuroinflammation after ischemic stroke induced by permanent distal middle cerebral artery occlusion followed by 1 h of hypoxia in a mouse model. The treatment of microglia from ischemic mice with TSW119, an inhibitor of GSK3β, significantly reduced the expression of pro-inflammatory proteins, such as CD16, TNFα, and iNOS and markedly increased the anti-inflammatory markers, such as CD206, IL-10, TGF-β, Arg-1, and YM1/2. Besides this, it contributed to the transition of microglia from the pro-inflammatory phenotype by reducing CD16/CD32^+^Iba^1+^ cells to an anti-inflammatory microenvironment in the peri-infract cortex by the reduction of TNF-α and the secretion of IL-10. These effects correlated with neurological improvement. This marked improvement of inflammation after the ischemic stroke was immediately reversed upon stimulation with IWR1, a specific inhibitor that targets Axin and leads to the degradation of β-catenin, suggesting that Wnt/β-catenin was able to attenuate inflammation after ischemic stroke. These data could suggest that Wnt/β-catenin activation during ischemic stroke could be a worthwhile therapeutic strategy to switch microglia polarization from a pro-inflammatory phenotype to a protective phenotype. After ischemic stroke, the transition of microglia polarization to an anti-inflammatory phenotype could be a strategy for inflammation amelioration by providing a better local microenvironment and neurovascular restoration (Song et al., 2019). In the same manner, renal ischemia reperfusion on mice treated with a Wnt agonist showed a significant decrease in IL-6 and IL-1β mRNA levels. Interestingly, this study demonstrates that the Wnt agonist is able to restore the proliferative ability of renal tubular cells by significantly increasing the number of Ki67 stained cells, illustrating a decrease of histologic injury and the amelioration of kidney architecture in general, including improvement in the abnormalities of Bowman's capsule spacing, hemorrhage, and intratubular cast formation (Kuncewitch et al., 2015a). Consistent with this, in a rat model for pulmonary hemorrhagic shock, the Wnt agonist reduced tissue damage by the reduction of neutrophil margination and cellular hyperplasia and the amelioration of edema and tissue infiltration. In addition, in the same model, the Wnt agonist showed its ability to preserve lung function by diminishing inflammatory cytokines in mRNA level (IL-6 and MPO) (Kuncewitch et al., 2015b). This suggests that the modulation of the Wnt pathway could be a strategy for the treatment of inflammatory disorders.

## Future Perspectives

In summary, recent studies suggest that Wnt molecules have a critical function in modulating responses of the immune system. One of its main activities seems to be to elicit regulatory and suppressive functions by the generation of tolerogenic DCs and influencing Treg cell and macrophage functions and inhibition of the largely pro-inflammatory NF-κB pathway. Hence, the Wnt pathway could serve as a molecular switch between opposing immune functions and could target different elements that may provide therapeutic benefits for various inflammatory diseases. On the other hand, a balance needs to be found when the inflammation spreads throughout the body because in systemic inflammation, Wnt signaling contributes to further disease. This underscores the complexity of Wnt signaling in immune cells, which is largely dose dependent in function (Luis et al., 2011; Staal et al., 2016). In fact, several Wnt inhibitors have already been shown to have a diverse mode of action ranging from blocking Fzd receptors and inhibiting Wnt secretion to the inhibition of β-catenin-mediated gene expression (Pai et al., 2017). However, these compounds lack cell type specificity and, in some cases, interact with both canonical and non-canonical Wnt signaling pathways. Besides this, none of these compounds have been sufficiently characterized for their efficacy, toxicity, and specificity in humans. Therefore, improvement of human target cell specificity might increase the chances for beneficial effects in various inflammatory disorders.

Even though Wnt signaling in DCs can clearly promote regulatory T cell responses and secrete cytokines in order to contain inflammation, there are remaining questions about the molecular mechanism and the downstream genes involved. In addition, the role of Wnt in both canonical and non-canonical pathways to regulate immunity and tolerance as well as the crosstalk between Wnt signaling and other pathways remains unsolved. Investigating expression levels of Wnt signaling components and their regulators in different inflammatory disease animal models is important in dissecting the dynamics of Wnt molecules in this setting. Besides this, performing loss-of-function studies (gene-deficient animals) as well as long-term overexpression of Wnt ligands is a promising approach that could help to reveal the long-term effect of these ligands in the remodeling process. In addition, unraveling the pathophysiological function of Wnt signaling components in an organ-specific manner by targeting tissue-specific expression using, for example, Cre-recombinase techniques would be useful. Such studies that yield another level of manipulating Wnt signaling allow the understanding of the exact inflammatory pathology and identification of therapeutic opportunities and also reveal potential risks and side effects of pharmacological interference with the Wnt signaling network.

## Author Contributions

IJ wrote the first draft, which was amended, completed and corrected by FS, KP-O, and KC-B. All authors saw and approved the final version.

## Conflict of Interest

The authors declare that the research was conducted in the absence of any commercial or financial relationships that could be construed as a potential conflict of interest.
